# Transvaginal versus transabdominal specimen extraction surgery for right colon cancer: A propensity matching study

**DOI:** 10.3389/fonc.2023.1168961

**Published:** 2023-04-06

**Authors:** Hongxin Yu, Weijie Lu, Chonghan Zhong, Houqiong Ju, Can Wu, Haocheng Xu, Dongning Liu, Taiyuan Li

**Affiliations:** Department of General Surgery, The First Affiliated Hospital of Nanchang University, Nanchang, Jiangxi, China

**Keywords:** transvaginal, natural orifice specimen extraction surgery, right colon cancer, transabdominal specimen extraction, survival

## Abstract

**Background:**

The transvaginal route for specimen extraction is considered ideal for colorectal surgery, but its safety is still questioned. There has been little research on transvaginal natural orifice specimen extraction surgery (NOSES) in the right hemicolectomy. As a result, we conducted a study comparing transvaginal NOSES to traditional transabdominal specimen extraction surgery.

**Patients and methods:**

Data on female patients who underwent radical right hemicolectomy at the First Affiliated Hospital of Nanchang University between January 2015 and December 2020 were collected retrospectively. A total of 847 patients were compliant, with 51 undergoing the transvaginal specimen extraction surgery (NOSES) group and 796 undergoing the transabdominal specimen extraction surgery (TISES) group. A propensity score matching method (1:2) was used to balance the clinicopathological characteristics of the two groups.

**Results:**

Finally, 138 patients were enrolled in our study, with 46 in the NOSES group and 92 in the TISES group. Compared to the TISES group, the NOSES group had less intraoperative blood loss (p = 0.036), shorter time to first flatus (p < 0.001), shorter time to first liquid diet (p < 0.001), lower postoperative white blood cell counts (p = 0.026), lower C-reactive protein levels (p = 0.027), and lower visual analog scale (VAS) scores (p < 0.001). Regarding the quality of life after surgery, the NOSES group had better role function (p < 0.01), emotional function (p < 0.001), and improved symptoms of postoperative pain (p < 0.001) and diarrhea (p = 0.024). The scar satisfaction was significantly higher in the NOSES group than in the TISES group. Overall survival and disease-free survival in two groups were similar.

**Conclusion:**

The short-term results of transvaginal NOSES were superior to conventional transabdominal specimen extraction surgery. At the same time, transvaginal NOSES could improve the abdominal wall appearance and quality of life. The long-term survival was similar in the two surgical approaches. Therefore, transvaginal NOSES is worthy of our implementation and promotion.

## 1 Introduction

Natural orifice specimen extraction surgery (NOSES), as a new and emerging technology, has developed rapidly in recent years, and its theoretical and technical framework has been continuously improved (1). NOSES removes the specimen through the natural cavity with only a few tiny postoperative scars. In the meantime, NOSES avoids the incisions produced by traditional surgery and the series of complications or effects derived from the incisions and demonstrates excellent minimally invasive results. Nowadays, NOSES is used in various fields such as general surgery, urology surgery, and gynecology (2–4).

Because of its excellent healing ability and ease of surgical operation, the vagina is considered the ideal route for specimen extraction of larger tumors and more highly located colorectal tumors (e.g., right hemicolectomy, 5). Compared to the transanal route, transvaginal specimen extraction for colorectal cancer is less common, and the safety and impact on sexual function are still questionable (6–8). However, transvaginal surgery has been performed in gynecology for a long time (9–11), Furthermore, its safety and feasibility have been recognized (12).

Currently, there are numerous clinical studies and meta-analyses on transanal NOSES, confirming that NOSES provides better short-term outcomes (13) and the long-term survival of patients undergoing NOSES is similar to those undergoing conventional surgery (14, 15). However, transvaginal NOSES is more complicated to perform. Few reports are available on transvaginal NOSES for right hemicolectomy. Therefore, we conducted this study to compare whether there is a difference in short-term outcomes and long-term survival between transvaginal specimen extraction and transabdominal specimen extraction in right hemicolectomy.

## 2 Methods

### 2.1 Patients

This study included 847 eligible female patients who underwent right hemicolectomy at the First Affiliated Hospital of Nanchang University between January 2015 and December 2020. Among them, 51 patients with transvaginal specimen extraction were allocated to the NOSES group, and 796 patients with transabdominal specimen extraction were allocated to the TISES group.

Inclusion criteria were (1) preoperative colonoscopy showing that the tumor was located in the ileocecal region, ascending colon, or transverse colon with pathology showing malignancy; (2) no distant metastasis; (3) American Society of Anesthesiologists (ASA) classification I, II, and III; (4) patients who signed informed consent; (5) patients, who were allocated to the NOSES group, without reproductive needs.

Exclusion criteria comprised (1) emergency surgery for intestinal obstruction and bleeding perforation; (2) a combination of primary malignancies or distant metastases from other organs; (3) incomplete data and missing follow-up data; (4) patients with a prophylactic stoma or other causes of a stoma; (5) preoperative radiotherapy; (5) combined organ resection; (6) body mass index (BMI) > 35 kg/m^2^.

### 2.2 Surgical procedure

After general anesthesia, a patient was placed in a functional lithotomy position, and the abdomen and vagina were disinfected with iodophor. The abdomen was first explored to determine the location of the tumor, the presence of metastasis, and other conditions. After severing the colonic mesentery along the ileocolic vessels, we entered the Toldts gap. The ileocecal artery and vein were then exposed, the root lymph nodes were cleared, and the ileocecal artery and vein were disconnected. The same method was used for the right colonic artery. The right hemicolon and its mesentery were freed after the right branch of the middle colonic artery was transected from the root and the surrounding lymph nodes were clear.

In the TISES group, a median abdominal incision was made. The intestinal canal was transected at the transverse colon (> 10 cm from the tumor) and 10 cm from the end of the ileum. Afterward, an end-to-end anastomosis between the transverse colon and ileum was performed, which was then reinforced.

**Figure 1:**
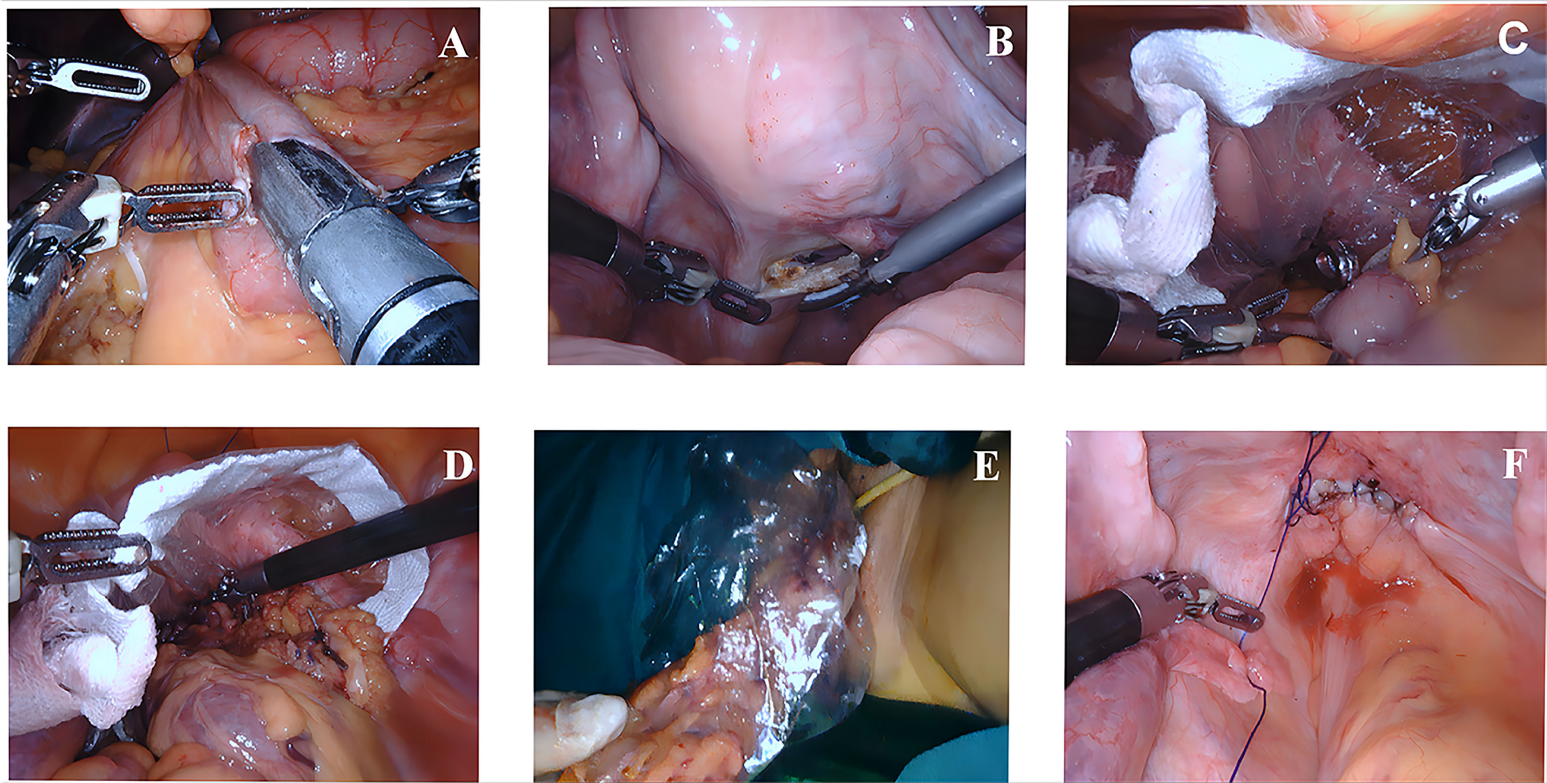
Key surgical steps of transvaginal specimen extraction surgery **(A–F)**. **(A)** The transverse colon and ileum were connected by side-to-side anastomosis. **(B)** The posterior vaginal wall was incised. **(C)** A sterile protective sleeve was placed into the vagina to establish sterile access. **(D, E)** Transvaginal specimen extraction. **(F)** Suturing the vaginal incision.

In the NOSES group, the terminal ileum was pulled up to the upper abdomen and placed parallel to the transverse colon. A side-to-side anastomosis between the transverse colon and ileum was performed with a linear stapler, and then the common opening was closed. The intestinal canal was transected at the transverse colon (> 10 cm from the tumor) and 10 cm from the end of the ileum. Afterward, the posterior vaginal wall was incised. A sterile protective sleeve was placed into the vagina to establish sterile access, and the specimen was dragged out transvaginally. Then, the vaginal incision was sutured. The critical operation for transvaginal specimen extraction is illustrated in **Figure 1**.

### 2.3 Data collection

All clinicopathological data from patients were collected, including age, BMI (kg/m^2^), tumor size (cm), carcinoembryonic antigen (CEA) levels, TNM stage (using the 8th edition of the AJCC TNM staging system for colorectal cancer), histological differentiation, and ASA classification. Additionally, perioperative indicators such as operating time, estimated intraoperative blood loss, postoperative hospital stay, postoperative complications, postoperative C-reactive protein (CRP) levels, postoperative white blood cell (WBC) counts, time to first flatus, time to first liquid diet, and visual analog scale (VAS) score were also recorded.

The Body Imagery Questionnaire (BIQ) includes the body image scale and cosmetic scales. Consequently, we used the body image scale to assess patients’ attitudes and satisfaction about their body appearance 2 months after surgery and the cosmetic scale to evaluate patients’ satisfaction with scar appearance. The EORTC QLQ-C30, which is a questionnaire from the European Organization for Research and Treatment of Cancer that assesses the quality of life, was used to evaluate the quality of life 2 months after surgery.

### 2.4 Follow-up

The follow-up method was similar to that shown by Tang et al. (16).

### 2.5 Statistical analysis

This study used propensity score matching (PSM) to balance the clinicopathological data between the two groups to reduce selection bias. We used Stata 17.0 software for 1:2 matching with a caliper value of 0.05. All statistical analyses were performed by the SPSS 25. Data conforming to normal distribution were expressed as mean ± standard deviation (SD) and tested by independent samples t-test. Non-normally distributed continuous data were expressed as median and range and tested by the Mann-Whitney U test. Categorical data were expressed as frequencies and percentages and tested by the χ^2^ test or Fisher exact probability. Disease-free survival and overall survival were compared by the log-rank test. Univariate survival analysis was performed using COX regression, and then variables with a P < 0.05 in the univariate survival analysis and those variables considered significantly correlated with survival in clinical work were selected for multivariate survival analysis. A P < 0.05 was considered statistically significant.

## 3 Results

### 3.1 Patients and clinicopathological characteristics

**Table 1** shows the comparison of clinicopathological data between the two groups before and after PSM. A total of 847 patients were enrolled in the study, including 51 patients in the NOSES group and 796 patients in the TISES group. The differences in BMI (24.05 ± 3.35 *vs.* 23.11 ± 2.84; p = 0.024), tumor size (4.21 ± 1.06 *vs.* 5.42 ± 2.28; p < 0.01), and infiltration depth (T stage, p < 0.01) between the NOSES and TISES groups were significant before PSM. After PSM (1:2), 46 patients were in the NOSES group, 92 patients were in the TISES group, and there was no statistical difference in the clinicopathological data between the two groups.

**Table 1:** Comparison of clinicopathological data of patients in two groups.

	Before PSM		p	After PSM		p
	NOSES(n=51)	TISES (n=796)		NOSES (n=46)	TISES (n=92)	
Age, mean(SD),years	60.27(12.67)	60.99(12.60)	0.694	58.96(12.57)	60.33(13.86)	0.574
Tumor size, mean(SD),cm	4.21(1.06)	5.42(2.28)	<0.001	4.12(1.09)	4.25(1.66)	0.635
BMI, mean(SD),kg/m^2^	24.05(3.35)	23.11(2.84)	0.024	24.00(3.48)	23.27(3.45)	0.223
T-stage, n (%)			<0.001			0.409
1	5(9.8)	36(4.5)		5(10.9)	11(12.0)	
2	5(9.8)	36(4.5)		4(8.7)	5(5.4)	
3	26(51)	212(26.6)		22(47.8)	34(37.0)	
4	15(29.4)	512(64.3)		15(32.6)	42(45.7)	
TNM stage, n(%)			0.077			0.550
I	8(15.7)	64(8.0)		8(17.4)	14(15.2)	
II	21(41.2)	430(54.0)		20(43.5)	33(35.9)	
III	22(43.1)	302(37.9)		18(39.1)	45(48.9)	
ASA, n(%)			0.223			0.889
I/II	12(23.5)	136(17.1)		11(23.9)	23(25.0)	
III	39(76.5)	670(82.9)		35(76.1)	69(75.0)	
CRP, mean(SD),mg/l	4.70(3.24)	4.35(2.67)	0.329	4.60(3.20)	4.89(2.67)	0.574
WBC, mean(SD),/l	5.38(1.22)	5.41(1.24)	0.823	5.41(1.25)	5.63(1.33)	0.356
CEA, n(%)			0.598			0.535
≤6.5	29(56.9)	483(60.7)		27(58.7)	59(64.1)	
>6.5	22(43.1)	313(39.3)		19(41.3)	33(35.9)	

BMI, body mass index; CRP, C-reactive protein; WBC, white blood cell; ASA, American Society of Anesthesiologists.

### 3.2 Intraoperative and postoperative outcomes

**Figure 2:**
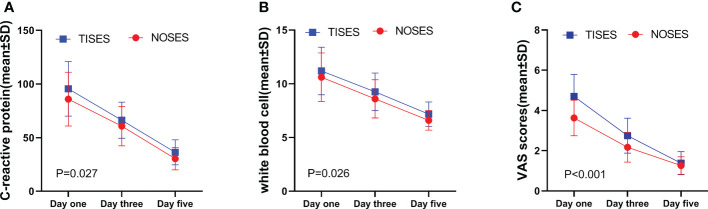
Comparison of postoperative CRP levels **(A)**, WBC **(B)**, and VAS score **(C)** in the two groups.

**Table 2** presents a comparison of the perioperative indexes between the two groups. Compared with the TISES group, the NOSES group had a longer operative time (164.00 ± 13.82 min *vs.* 146.90 ± 17.38 min; p < 0.001), faster postoperative gastrointestinal recovery (55.98 ± 8.84 h *vs.* 64.76 ± 9.25 h; p < 0.001), and earlier postoperative fluid diet (64.89 ± 7.70 h *vs.* 72.93 ± 8.50 h; p < 0.001). Postoperative hospital stay and complication rates were comparable in both groups. However, four incisional infections occurred in the TISES group (**Table 2**). Concerning postoperative pain scores, the NOSES group had significantly lower VAS scores than the TISES group after surgery (p < 0.001, **Table 3**). In the comparison of postoperative inflammatory reaction, WBC (p = 0.026) and CRP (p = 0.027) were significantly lower in the NOSES group than in the TISES group (**Table 3**, **Figure 2**).

**Table 2:** Perioperative indexes between the NOSES group and the TISES group.

	After PSM		P
	NOSES(n=46)	TISES(n=92)	
Operation time (mean ± SD), min	164.00(13.82)	146.90(17.38)	<0.001
Intraoperative blood loss (mean ± SD), ml	71.20(22.34)	80.76(26.15)	0.036
Time to first flatus (mean ± SD), h	55.98(8.84)	64.76(9.25)	<0.001
Time to first liquid diet (mean ± SD), h	64.89(7.70)	72.93(8.50)	<0.001
Postoperative hospital stay (mean ± SD), d	7.78(2.24)	8.73(3.53)	0.1
Postoperative complications,n(%)	5(10.87)	13(14.13)	0.592
Anastomotic leakage	0(0)	1(1.09)	
Wound infection	0(0)	4(4.35)	
Ileus	1(2.17)	2(2.17)	
Pulmonary infection	1(2.17)	2(2.17)	
Abdominal infection	1(2.17)	1(1.09)	
Anastomotic bleeding	1(2.17)	1(1.09)	
Urinary infection	1(2.17)	1(1.09)	
Gastroplegia	0(0)	1(1.09)	

**Table 3:** Comparison of VAS scores, WBC and CRP levels between the NOSES group and the TISES group.

	After PSM		P
	NOSES(n=46)	TISES(n=92)	
Postoperative CRP, mean (SD)			0.027
Day one	85.96(25.06)	95.62(25.49)	
Day three	60.72(18.45)	66.24(16.89)	
Day five	30.41(10.42)	36.36(11.71)	
Postoperative WBC, mean (SD)			0.026
Day one	10.61(2.26)	11.19(2.22)	
Day three	8.60(1.78)	9.25(1.75)	
Day five	6.59(0.91)	7.18(1.14)	
VAS score, mean (SD)			<0.001
Day one	3.63(0.88)	4.70(1.10)	
Day three	2.17(0.74)	2.75(0.87)	
Day five	1.26(0.44)	1.38(0.57)	

Using repeated measures statistical analysis to calculate the P-value. Day one, the first day after surgery; day three, the third day after surgery; day five, the fifth day after surgery.

### 3.3 The comparison of the postoperative pathology between the two groups

**Table 4** shows the comparison of the postoperative pathological findings between the two groups. There were no statistical differences between the NOSES and TISES groups regarding the number of lymph nodes harvested (p = 0.784), N stage (p = 0.820), perineural invasion (p = 0.806), lymphatic or vascular invasion (p = 0.789), and histological differentiation (p = 0.843, **Table 4**). It indicated that NOSES could achieve a similar degree of tumor eradication as TISES.

**Table 4:** Comparison of the postoperative pathological findings between the NOSES group and the TISES group.

	After PSM		
	NOSES(n=46)	TISES(n=92)	P
Number of lymph nodes harvested, mean (SD)	22.82(13.69)	22.34(7.44)	0.784
N-stage, n (%)			0.820
0	28(60.9)	51(55.4)	
1	11(23.9)	26(28.3)	
2	7(15.2)	15(16.3)	
Perineural invasion, n (%)			0.806
+	18(39.1)	38(41.3)	
-	28(60.9)	54(58.7)	
Lymphatic or vascular invasion, n (%)			0.798
+	16(34.8)	30(32.6)	
-	30(65.2)	62(67.4)	
Histological differentiation, n (%)			0.843
well	2(4.3)	6(6.5)	
Moderate	36(78.3)	72(78.3)	
Poor	8(17.4)	14(15.2)	

### 3.4 Comparison of quality of life between the NOSES group and the TISES group

**Figure 3:**
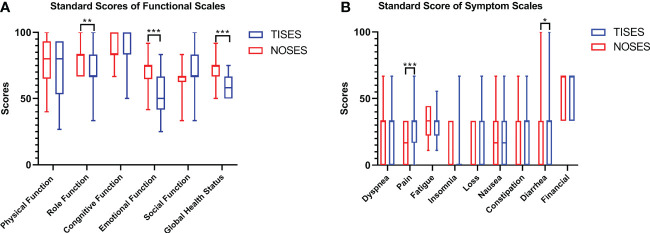
Comparison of EORTC QLQ-C30 results [**(A)** standard scores of functional scales, **(B)** standard score of symptom scales] between the two groups. *p < 0.05, **p < 0.01, ***p < 0.001. The p-value was calculated by the Mann-Whitney U test.

**Figure 4:**
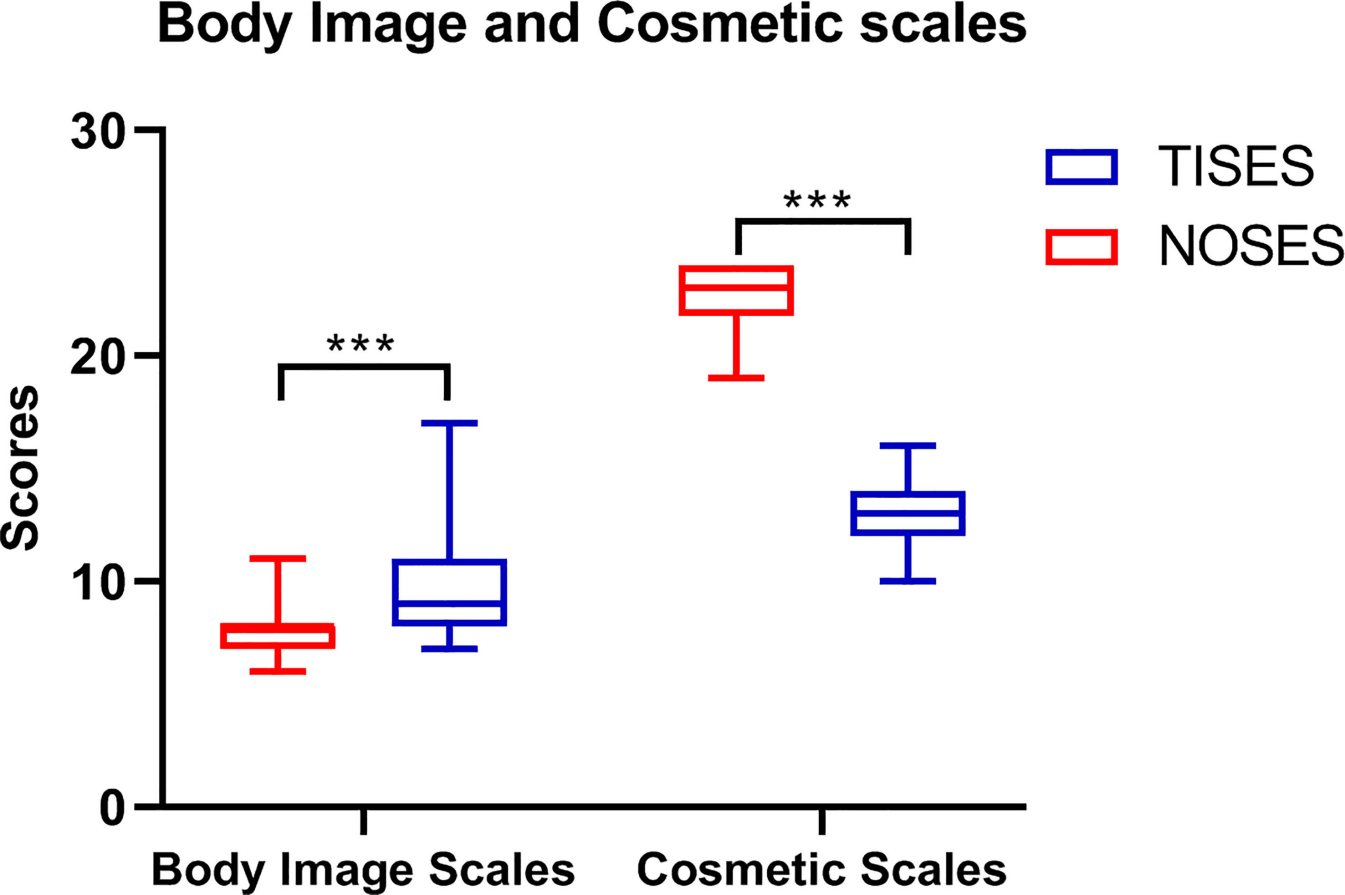
Comparison of Body Image Questionnaire (BIQ) in two groups. ***p < 0.001.

In this study, the EORTC QLQ-C30 was used to assess the patients’ quality of life 2 months after surgery. In function scales, higher scores meant better function. In symptom scalds, the lower scores meant better symptoms. Compared to the TISES group, the NOSES group had better role function (p < 0.01), global health status (p < 0.001), and emotional function (p < 0.001, **Figure 3A**). Regarding postoperative symptom scores, the NOSES group was significantly superior to the TISES group in terms of postoperative pain (p < 0.001) and diarrhea (p < 0.05, **Figure 3B**). In the BIQ scores 2 months after surgery, the NOSES group had significantly lower body image scores (p < 0.001) and higher cosmetic scores (p < 0.001) than the TISES group (**Figure 4**).

### 3.5 Long-term survival outcomes between the two groups

**Figure 5:**
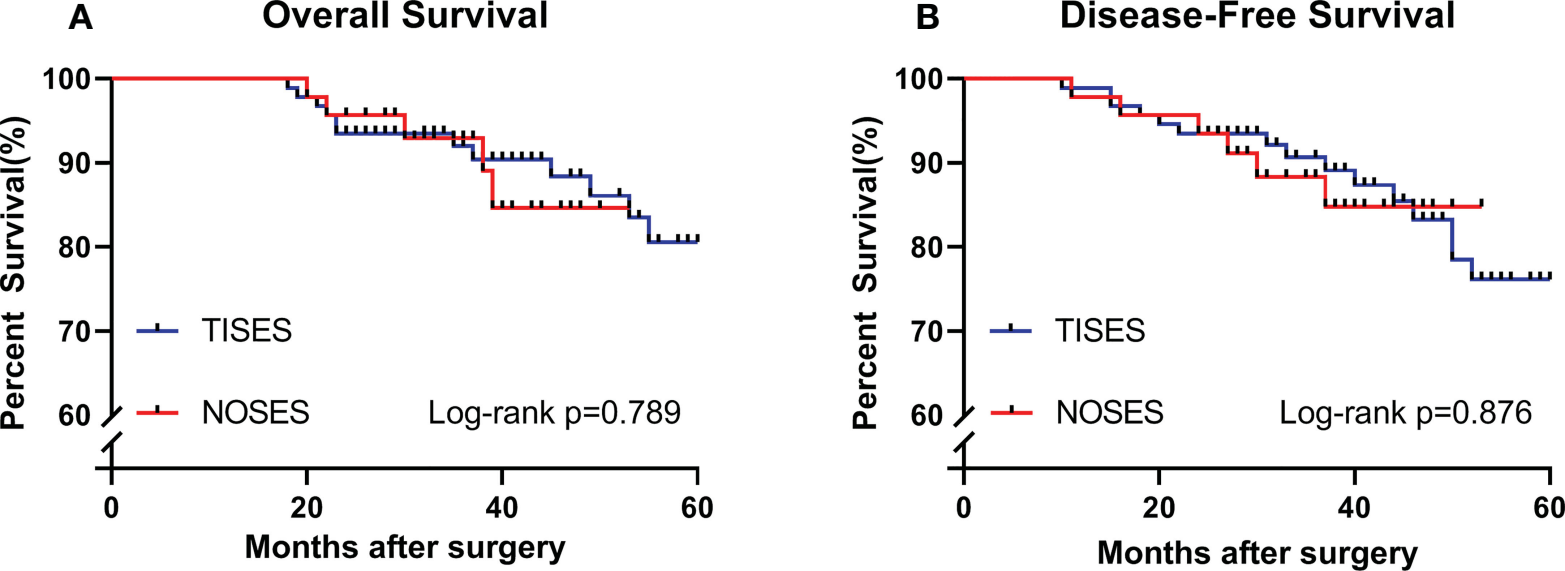
Comparing the survival curves between the two groups in terms of overall survival **(A)** and disease-free survival **(B)**. P-value was calculated by log-rank test.

As of 2020-Dec, the median follow-up time was 38 months (20–53) in the NOSES group and 42 months (18–60) in the TISES group. In this study, the maximum period of follow-up was 60 months (patients beyond 60 months postoperatively were not followed up further). Three-year overall survival in the NOSES group was 92.9%, and 3-year overall survival in the TISES group was 91.2%. **Figure 5** shows the survival analysis of the NOSES group *vs.* the TISES group. There were no statistical differences in overall survival (log-rank p = 0.789) and disease-free survival (log-rank p = 0.876) between the two groups.

### 3.6 Univariate and multivariate survival analysis of overall and disease-free survival

**Tables 5** and 6 depict the univariate and multivariate survival analysis of overall survival and disease-free survival. The univariate overall survival analysis in **Table 5** showed that T-stage (p = 0.047), lymphatic or vascular invasion (p = 0.001), and N-stage (p < 0.001) were associated with overall survival. The univariate disease-free survival analysis in **Table 6** showed that perineural invasion (p = 0.021), lymphatic or vascular invasion (p < 0.001), and N-stage (p < 0.001) were associated with disease-free survival. Multifactorial overall survival analysis showed that the N-stage (p < 0.001) was associated with overall survival. Multifactorial disease-free survival analysis showed that the N stage (p = 0.001) and lymphatic or vascular invasion (p = 0.030) were associated with disease-free survival. Therefore, we consider the N-stage an independent prognostic factor for overall survival and disease-free survival.

**Table 5:** Univariate and multivariate survival analysis of overall survival.

Univariate survival analysis of overall survival				
	n	HR	95% CI	p
Operation		1.181	0.399–3.496	0.764
NOSES	46			
TISES	92			
Age	136	0.995	0.960–1.032	0.791
Tumor size	136	1.043	0.760–1.430	0.796
T-stage	136	2.319	1.010–5.326	0.047
Lymphatic or vascular invasion		5.307	1.928–14.604	0.001
-	92			
+	46			
Perineural invasion		2.620	0.968–7.088	0.058
-	82			
+	56			
N-stage	136	5.008	2.453–10.224	<0.001
Multivariate survival analysis of overall survival				
	n	HR	95% CI	p
T-stage	136	2.335	0.861–6.331	0.096
Lymphatic or vascular invasion	136	2.724	0.918–8.085	0.071
Perineural invasion	136	0.347	0.102–1.177	0.089
N-stage	136	5.848	2.287–14.950	<0.001

HR, hazard ratio; 95% CI, 95% confidence interval.

**Table 6:** Univariate and multivariate survival analysis of disease-free survival.

Univariate survival analysis of disease-free survival				
	n	HR	95% CI	p
Operation		1.094	0.414–2.889	0.856
NOSES	46			
TISES	92			
Age	136	0.999	0.967–1.033	0.967
Tumor size	136	1.030	0.777–1.365	0.837
T-stage	136	1.715	0.943–3.118	0.077
Lymphatic or vascular invasion		5.729	2.287–14.350	<0.001
-	92			
+	46			
Perineural invasion		2.906	1.173–7.201	0.021
-	82			
+	56			
N-stage	136	4.110	2.281–7.405	<0.001
Multivariate survival analysis of disease-free survival				
	n	HR	95% CI	p
T-stage	136	1.616	0.780–3.348	0.196
Lymphatic or vascular invasion	136	2.966	1.110–7.929	0.030
Perineural invasion	136	0.520	0.166–1.634	0.263
N-stage	136	3.951	1.816–8.594	0.001

HR, hazard ratio; 95% CI, 95% confidence interval.

## 4 Discussion

In recent decades, the surgical treatment of colorectal cancer has advanced rapidly. From open surgery to laparoscopic surgery and emerging NOSES and robotic surgery, technological advances have improved patient outcomes. In general surgery, transvaginal gallbladder extraction was first reported in 1993 (17), In 1996, Redwine et al. (18) attempted laparoscopic-assisted resection of benign colorectal disease and transvaginal specimen extraction surgery. Subsequently, transvaginal specimen extraction was used for colorectal and gastric cancers (19). The vagina is very flexible and can accommodate larger volumes, while the incision and suturing in the posterior vaginal vault, which has great healing results, have a low probability of fistula and infection. Therefore, for women who do not require fertility, the vaginal route is the ideal option for specimen removal.

The operative time in our research was obviously longer in the NOSES group than in the TISES group. The NOSES group required incision and suturing of the vagina, intra-abdominal anastomosis, and reinforcement of the anastomosis. Additionally, this was a challenge for the operator, and the operation was longer. However, the NOSES group had faster postoperative recovery of gastrointestinal function (20). Ron Shapiro et al. have considered that intra-abdominal anastomosis is less straining for the colon and has a lower risk of mesenteric tears, bleeding, and tissue torsion; therefore, NOSES was more conducive to the recovery of gastrointestinal function (21). Although the procedure was longer, the extra time spent was worth it for the rapid recovery and higher quality of life after NOSES.

On the one hand, the abdominal incision is the most direct indicator of the minimally invasive effect of the surgery. The surgical incision is the main factor causing postoperative pain and also one of the factors influencing postoperative recovery. Simultaneously, abdominal incisions increase the risk of complications such as incisional infection, incisional hernia, and tumor implantation (22). In our study, the postoperative pain scores were obviously lower in the NOSES group than in the TISES group. Additionally, a total of four wound infections were seen in 138 patients, all of which were in the TISES group. On the other hand, we could assess not only the physical impact of the incision but also the psychological impact, which is equally important. Pain and abnormal sensations of postoperative scarring can bring negative psychological and adverse emotions to patients (13). Abdominal scarring not only affects the aesthetics of the abdominal wall but may also impose limitations on life, recreation, and work. All of these factors can have an impact on the psychological health of the patient. At the postoperative follow-up, the NOSES group was superior to the TISES group in role function, emotional function, and global health status. In terms of postoperative symptom scores, the NOSES group had much fewer pain and diarrhea symptoms. Furthermore, patients who received transvaginal NOSES had better satisfaction with the scar and abdominal beauty.

Regarding surgical safety, there were 5 complications in the NOSES group and 13 in the TISES group, with no statistical difference. For the risk of potential abdominal infection in NOSES, In our present research, there was one case of abdominal infection in the NOSES group and one in the TISES group. The probability of developing an abdominal infection in a Chinese NOSES study that included 5055 patients was 1.9% (23).

Therefore, we believe that NOSES does not increase the risk of abdominal infection in the presence of strict aseptic principles. With reference to whether transvaginal specimens may affect the patient’s sexual function or cause other vaginal-related complications, complications such as vaginal fistula or infection were not found in our research, and some studies have reported similar results (24, 25). In transvaginal NOSES, our chosen vaginal incision site was the posterior vaginal vault, which is not surrounded by important nerves and does not produce arousal to sexual stimulation. Sexual function was affected for a short time after transvaginal NOSES, and the procedure did not increase the risk of sexual dysfunction in the long term in Zheng et al.’s research (26). In terms of tumor eradication, there were no differences in the number of lymph nodes harvested, N-stage, perineural invasion, lymphatic or vascular invasion, and histological differentiation between NOSES and TISES, suggesting that the same degree of eradication can be achieved with NOSES, which is similar to results reported by Kim et al. (27).

From our postoperative follow-up, the overall survival and disease-free survival of NOSES and TISES were similar, which is similar to the results reported by Li et al. (28). In the univariate cox regression analysis, there was no statistical difference in the two surgical approaches for either disease-free survival or overall survival, and this was consistent with the findings of Liu et al. (29). This demonstrated that different surgical approaches have no obvious impact on patient’s survival when radical resection of the tumor has been achieved. Combining the results of univariate and multifactorial COX regression, we considered that the most important factor affecting the long-term survival of patients was the N-stage.

It is important to acknowledge that this study had some limitations. First, our study was retrospective research; therefore, selection bias were inevitable. Thus, we used PSM to reduce the difference between the two groups. Second, transvaginal extraction of the specimen after right hemicolectomy is a difficult procedure, limiting its use in clinical practice. Meanwhile, the study was a single-center study; hence, the sample size was not large, which might have made the results of our COX regression less accurate. We expect more prospective, large-sample, multicenter randomized controlled studies to provide better evidence-based medical help.

## 5 Conclusion

Overall, transvaginal NOSES is a safe and feasible technique for treating patients with right colon cancer. Transvaginal NOSES had better short-term outcomes than transabdominal specimen extraction surgery, such as less intraoperative bleeding and faster recovery of gastrointestinal function. Transvaginal NOSES provided a better postoperative quality of life and scar satisfaction. Meanwhile, the long-term survival was similar in the two surgical approaches. The N-stage was probably the major factor affecting long-term survival.

## Data availability statement

The raw data supporting the conclusions of this article will be made available by the authors, without undue reservation.

## Ethics statement

Written informed consent was obtained from the individual(s) for the publication of any potentially identifiable images or data included in this article.

## Author contributions

All authors listed have made a substantial, direct contribution to the work and approved it for publication. All authors contributed to the article and approved the submitted version.
